# Acquisition of N-Glycosylation Sites in Immunoglobulin Heavy Chain Genes During Local Expansion in Parotid Salivary Glands of Primary Sjögren Patients

**DOI:** 10.3389/fimmu.2018.00491

**Published:** 2018-03-12

**Authors:** Annie Visser, Marieke E. Doorenspleet, Niek de Vries, Fred K. L. Spijkervet, Arjan Vissink, Richard J. Bende, Hendrika Bootsma, Frans G. M. Kroese, Nicolaas A. Bos

**Affiliations:** ^1^Department of Rheumatology and Clinical Immunology, University of Groningen and University Medical Center Groningen, Groningen, Netherlands; ^2^Department of Clinical Immunology and Rheumatology, Academic Medical Center and University of Amsterdam, Amsterdam, Netherlands; ^3^Rheumatology and Immunology Center, Academic Medical Center, Amsterdam, Netherlands; ^4^Laboratory for Genome Analysis, Academic Medical Center, Amsterdam, Netherlands; ^5^Department of Oral and Maxillofacial Surgery, University of Groningen and University Medical Center Groningen, Groningen, Netherlands; ^6^Department of Pathology, Academic Medical Center and University of Amsterdam, Amsterdam, Netherlands

**Keywords:** Sjögren syndrome, B-cell, N-glycosylation, heavy chain, parotid Gland, next generation sequencing

## Abstract

Previous studies revealed high incidence of acquired N-glycosylation sites acquired N-glycosylation sites in RNA transcripts encoding immunoglobulin heavy variable region (IGHV) 3 genes from parotid glands of primary Sjögren’s syndrome (pSS) patients. In this study, next generation sequencing was used to study the extent of ac-Nglycs among clonally expanded cells from all IGVH families in the salivary glands of pSS patients. RNA was isolated from parotid gland biopsies of five pSS patients and five non-pSS sicca controls. IGHV sequences covering all functional IGHV genes were amplified, sequenced, and analyzed. Each biopsy recovered 1,800–4,000 unique IGHV sequences. No difference in IGHV gene usage was observed between pSS and non-pSS sequences. Clonally related sequences with more than 0.3% of the total number of sequences per patient were referred to as dominant clone. Overall, 70 dominant clones were found in pSS biopsies, compared to 15 in non-pSS. No difference in percentage mutation in dominant clone-derived IGHV sequences was seen between pSS and non-pSS. In pSS, no evidence for antigen-driven selection in dominant clones was found. We observed a significantly higher amount of ac-Nglycs among pSS dominant clone-derived sequences compared to non-pSS. Ac-Nglycs were, however, not restricted to dominant clones or IGHV gene. Most ac-Nglycs were detected in the framework 3 region. No stereotypic rheumatoid factor rearrangements were found in dominant clones. Lineage tree analysis showed in four pSS patients, but not in non-pSS, the presence of the germline sequence from a dominant clone. Presence of germline sequence and mutated IGHV sequences in the same dominant clone provide evidence that this clone originated from a naïve B-cell recruited into the parotid gland to expand and differentiate locally into plasma cells. The increased presence of ac-Nglycs in IGHV sequences, due to somatic hypermutation, might provide B-cells an escape mechanism to survive during immune response. We speculate that glycosylation of the B-cell receptor makes the cell sensitive to environmental lectin signals to contribute to aberrant B-cell selection in pSS parotid glands.

## Introduction

Primary Sjögren’s syndrome (pSS) is clinically characterized by complaints of dry mouth and dry eyes (sicca complaints), which are associated with the presence of periductal lymphoid infiltrates in the salivary and lacrimal glands. From a pathogenic point of view, B-cell hyperactivity is a hallmark of the disease (1). This is reflected by the presence of elevated serum levels of IgG in patients with pSS, as well as by the presence of autoantibodies, such as anti-Ro/SSA and anti-La/SSB autoantibodies, and rheumatoid factor (RF) (2, 3).

The periductal infiltrate of the salivary glands harbors many B-cells, which even can form ectopic germinal centers (GCs), and there is a profound increase in the number of IgG plasma cells (4). Mucosa-associated lymphoid tissue (MALT) lymphomas develop frequently in the parotid glands of patients with pSS (1, 5). B-cell hyperactivity is further revealed by the presence of clonal populations of B-cells and plasma cells in the minor (labial) and major (parotid) salivary gland tissue of pSS patients (6–10). Nearly all IgG and IgA encoding genes derived from clonally related cells contain somatic mutations and, thus, are thought to originate from post-GC memory B-cells and plasma cells (9–11). The reason for the considerable clonal expansion of B-cells in pSS is not known, but proliferation and survival mediated by enhanced signaling through the B-cell receptor (BCR) may be involved. This presumption is in line with the observation that both naïve and memory B-cells in peripheral blood of pSS patients express increased levels of Bruton’s tyrosine kinase, a molecule that is critically involved in BCR signaling (12). The elevated levels of B-cell associated cytokines, such as BAFF, APRIL, IL-6, and IL-21 present in serum and saliva of these patients, may further support the development and persistence of these clonal B-cell and plasma cell populations (13–21).

In our previous study (10), the analysis of the mutation patterns of the immunoglobulin heavy chain variable 3 genes (IGHV3) revealed no evidence that antigen selection plays a major role in clonal B-cell expansions in the parotid gland of pSS patients. This observation may indicate that alternative driving forces and selection pressures are active in these clonal expansions. One such driving force might be the presence of newly acquired sugar moieties of the immunoglobulins expressed by B-cells as has been shown for follicular lymphoma (22–24). These lymphomas more frequently show acquired N-glycosylation sites (ac-Nglycs), within the variable domain of tumor-specific immunoglobulins. N-linked glycosylation requires the consensus amino acid (AA) motif N-X-S/T (asparagine-X-serine/threonine). We observed a higher prevalence of these ac-Nglycs in the IGHV3 encoding transcripts derived from IgG (but not in IgA) expressing clones in the parotid gland of pSS patients compared to non-pSS sicca controls. Surprisingly, most (>60%) of these ac-Nglycs were situated in the framework (FWR) 3 region and not in the complementarity determining regions (CDRs) of the immunoglobulin heavy variable region (IGHV) sequences. Ac-Nglycs are not only increased in pSS but also in other rheumatoid and non-rheumatoid autoimmune diseases as systemic lupus erythematosus (SLE), multiple sclerosis, chronic Chagas’ heart disease, and RA (25). In RA, there is an increase of ac-Nglycs in the immunoglobulin variable (V) region heavy and light chain transcripts from anti-citrullinated protein antibody IgG-expressing B-cells (26).

Our previous finding that ac-Nglycs are enriched in IGHV3 transcripts from clonal expansions of Ig-producing cells in parotid glands of pSS patients may point toward a selective advantage for B-cells to expand and survive. A question that arises is whether these ac-Nglycs are unique for IGHV3 genes or are also seen in IGHV genes from other IGHV families. Furthermore, it remains to be shown whether ac-Nglycs are restricted to clonally expanded B-cells and plasma cells, and to what extent BCRs with ac-Nglycs encode for autoantibodies, such as stereotypic RF. Finally, it has to be unraveled whether naïve and/or memory B-cells proliferate and expand to form clones within the microenvironment of the salivary gland tissue of pSS patients. Here, we utilize next-generation sequencing (NGS) to address the above issues regarding clonal expansion of Ig-producing cells in pSS patients.

## Materials and Methods

### Patients

Parotid biopsies of five pSS patients fulfilling the ACR-EULAR (27) criteria for pSS (all female; median age 55 years, range 18–80 years), with clinically a disease duration of less than 5 years were included for this study. Other inclusion criteria for pSS patients in this study were as follows: stimulated whole saliva secretion flow >0.15 ml/min, presence of autoantibodies (ANA positive, IgM-RF ≥ 10 klU/l, or presence of anti-SSA/anti-SSB autoantibodies).

In addition, five parotid biopsies of patients with sicca complaints were included as non-pSS sicca controls (all females; median age 66 years, range 59–75 years). These non-pSS sicca controls have the same subjective complaints of dry mouth and dry eyes as pSS patients, but they do not fulfill the ACR-EULAR criteria for pSS. A biopsy of the parotid gland under local anesthesia was taken as part of the clinical diagnostic work-up for pSS (28). Histopathological examination of the parotid glands of all five control patients revealed normal histology of the glandular tissue. After analysis of all data, we excluded non-pSS sicca control 2 because we observed a large clone of >640 members (31% of the total non-pSS sequences). Because we could not rule out the presence of malignancies in the parotid gland, or the presence of lymph nodular tissue, this patient was excluded from the study.

Parotid biopsies were originally collected for diagnostic purposes. Usage of these biopsies for research purposes was obtained on written informed consent with approval from the medical ethical committee of the University Medical Center Groningen, Groningen, the Netherlands.

### RNA Isolation and cDNA Synthesis

Parotid gland biopsies were snap-frozen in liquid nitrogen after surgery and cryo-preserved at −80°C until use. RNA was isolated from parotid gland samples using a polytron tissue homogenizer (Kinematica AG, Littau-Lucerne, Switzerland) in the presence of STAT60 RNA reagent (Tel-test Inc., Friendswood, TX, USA) according to the manufacturer’s protocol. After isolation RNA was purified using the RNeasy Mini System [Clean-up-protocol (#74106, Qiagen, Venlo, the Netherlands)]. RNA quality was checked using the Bioanalyzer 2100 system (Agilent) and quantified using the Qubit1.0-platform (#Q32857, Invitrogen Life Technologies, Breda, the Netherlands). cDNA was synthesized using Superscript RT-III and oligo-dT primers according to the manufacturer’s protocol (#18080-051, Invitrogen).

### Linear Amplification and NGS

Linear amplification of the IGHV genes was based on the protocol used previously (29, 30) using primer sets (available upon request) that covered all functional IGHV genes. Briefly, the IGHV region primers contained a primer B sequence required for amplicon sequencing (Roche Diagnostics, Mannheim, Germany). Amplified products were purified and used in a generic PCR using primer B as forward primer and a reverse generic primer specific for all functional IGHJ genes, containing primer sequence A.

Samples were again purified, quantified, prepared for sequencing according to the manual for amplicon sequencing, and sequenced on a Roche Genome Sequencer FLX (titanium platform).

The bioinformatics pipeline used to obtain the IGHV sequences was done by performing Multiplex Identifier sorting, identification of gene segments, and removal of artifacts.

### Analysis of Rearranged Immunoglobulin Genes

The IGHV sequences were analyzed for IGHV gene usage, rearrangement, and mutations by aligning them with the human Ig set of the IMGT reference directory.^1^ The V-region, D-region, and J-region alleles closest to the reference germline Ig sets were assigned to the obtained IGHV sequence. Only productive sequences (encoding functional proteins) were included in this study. IGHV sequences that were out of frame, too short to assign junction analysis or sequences with insertions or deletions were discarded. One hundred percent identical IGHV sequences obtained from a single biopsy were counted as one, because it is not possible to discriminate between sequences derived from multiple transcripts of the same cell or identical sequences derived from different cells. Unique sequences were selected based upon ≥1 nucleotide difference.

Immunoglobulin heavy variable region sequences with identical nucleotide sequences at the CDR3, and shared the same VH germline gene, were considered as clonally related. Together clonally related sequences form a “clone.” Clones were considered dominant when the number of clonally related sequences was ≥0.3% of the total number of unique recovered sequences per patient (arbitrarily chosen) of the total number of productive sequences obtained from a single biopsy.

To examine whether the IGHV sequences encode for stereotypic RF BCRs, we analyzed the IGHV sequences for the presence of stereotypic RF VH/JH rearrangements, combined with VH-CDR3 AA sequence homology to stereotypic RF VH-CDR3 AA sequences (11, 31, 32). Screening for VH-CDR3 AA homology was performed using the NCBI Protein-BLAST algorithm (search for short nearly exact matches).^2^ Criteria used for homology were ≥60% AA homology and a length difference between the VH-CDR3 sequences not exceeding three AA (32).

### Analysis of Clonal Expansion

To gain understanding of the origin of the clonal expansion of B-cells in the parotid gland, lineage trees were created from clonally related IGHV sequences using the algorithm of the IgTree© (33). Briefly, IGHV sequences from a dominant clone together with the germline sequence were aligned using ClustalW software.^3^ The output alignment was used as input in IgTree©. The output tree file is visualized by Graphviz 2.38. The root of the tree is the putative germline sequence and the clonally related IGHV sequences are assigned to either leaves or internal nodes of the tree. Each tree node represents a single mutation separating the sequences. Missing sequences are artificially added by IgTree©.

### Analysis of Selection Pressures

Selection pressure analysis of the IGHV sequences belonging to a dominant clone was studied using the online Bayesian Estimation of Antigen-Driven Selection in Immunoglobulin Sequences (BASELINe) program. This program uses statistical algorithms based on analysis of somatic mutation patterns of the IGHV sequences to predict the selection pressure which shape the IGHV repertoire of Ig-producing cells. The distribution of replacements versus silent (R/S) mutations, in both CDRs and FWRs is counted separately and compared against the expected frequency. BASELINe aggregates the selection strength of different sequences within a single experimental group and to compare the selection pressures between different experimental groups (34).

### Prediction of Acquired N-Glycosylation Sites

Ac-Nglycs were predicted on the basis of the translated AA sequences of the IGHV sequences (including the CDR3 region) using the NetN-glyc 1.0 online server.^4^ The server uses artificial neural networks to examine the context of the N-X-S/T (asparagine-X-serine/threonine) motif in the IGHV sequences. We used the restriction that X could be any AA except proline, as it precludes N-glycosylation due to steric hindrance. Criteria for accepting ac-Nglycs used were as follows: potential >0.5 and jury agreement ≥5/9. The ability of the software program to predict actual N-glycosylation sites has an overall accuracy of 76%.

### Statistical Analysis

All statistical analyses were performed using GraphPad Prism software (version 3.0). Statistical comparisons of data from pSS patients and non-pSS sicca controls were carried out using Mann–Whitney *U* test. *p* Values <0.05 were considered as statistically significant.

## Results

### No Major Differences Found in VH-Gene Usage of Ig-Producing Cells in Parotid Glands of pSS and Non-pSS Sicca

A total of 15.756 unique IGHV sequences were collected from five parotid biopsies of pSS patients (1.815–4.039 per biopsy) and 11.834 unique IGHV sequences in the 4 biopsies of non-pSS sicca controls (2.673–3.191 per biopsy).

**Figure 1:**
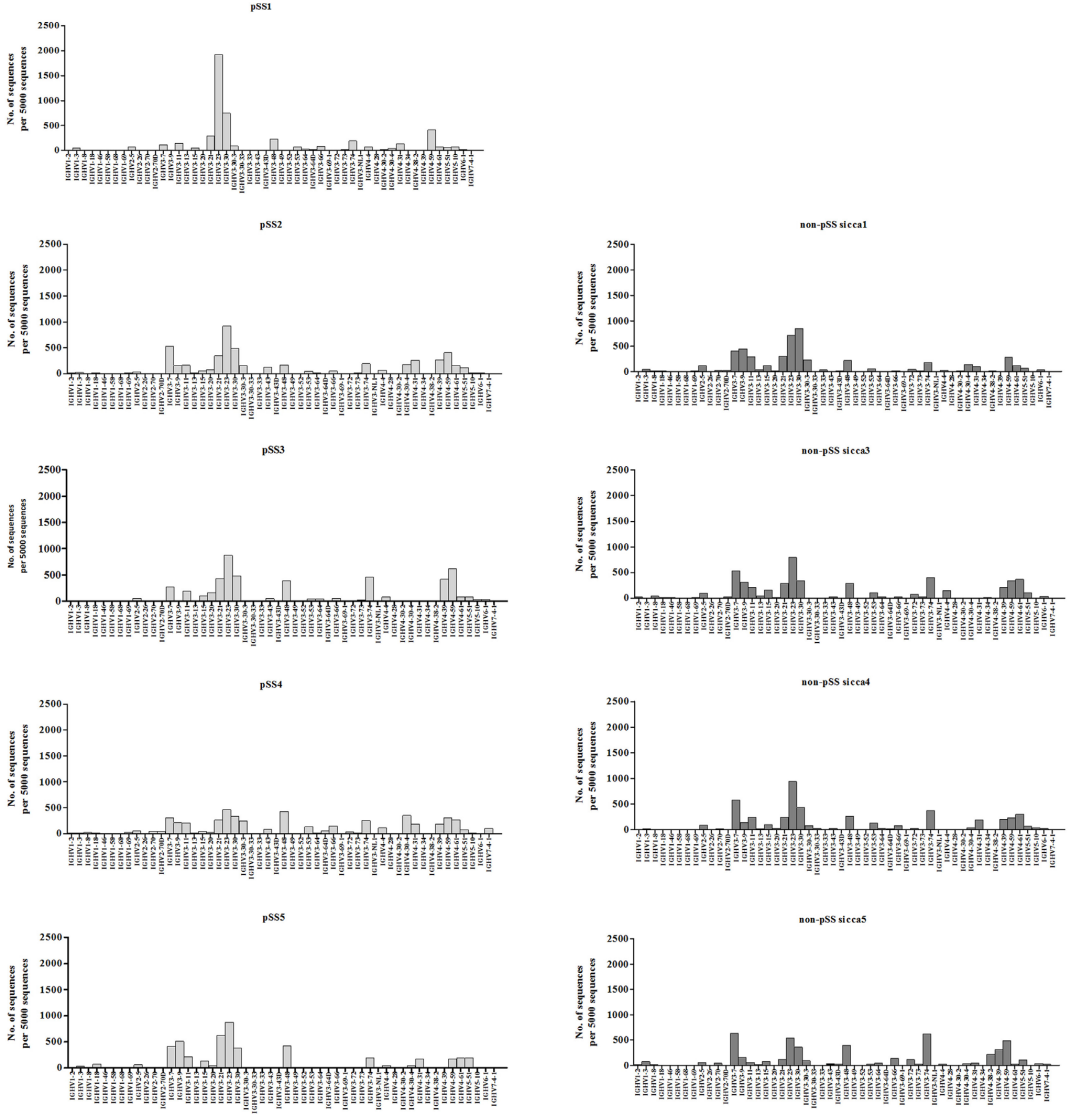
Percentage of IGVH gene usage in the parotid gland biopsies of pSS patients compared to those from non-pSS sicca controls.

No significant differences in the usage of various VH genes among the IGHV sequences derived from pSS and non-pSS sicca biopsies were observed except for VH3-72 (Figure 1). The latter was significantly (*p* = 0.0268) more frequently used in non-pSS sicca (median 1.17% IQR; 0.64–2.07%) than in pSS (median 0.18% IQR; 0.08–0.40%) biopsies. The CDR3 lengths of the IGHV sequences between the two patient groups were also similar (data not shown).

### Higher Frequency of Germline IGHV Sequences in Parotid Glands of pSS Patients

**Figure 2:**
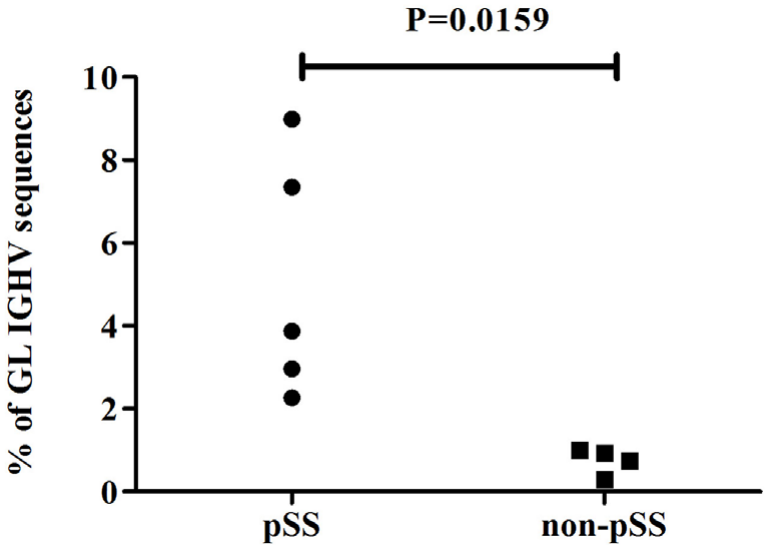
Percentage of germline (≤1 nucleotide difference from GL gene) encoded IGHV sequences in parotid gland biopsies of pSS patients and non-pSS sicca controls.

Next, we analyzed the presence of somatic hypermutations among the IGHV sequences amplified from cDNA of parotid glands of individual pSS patients and non-pSS sicca controls. IGHV sequences with ≥1 nucleotide difference with the closest reference germline gene were considered to be mutated as a consequence of somatic hypermutation. A significant (*p* = 0.0159) higher percentage of germline encoded IGHV sequences was found among sequences obtained from the parotid gland of pSS patients (Figure 2; pSS median 3.9% IQR: 2.6–8.2%, non-pSS sicca median 0.8% IQR: 0.4–1.0). The higher percentage of germline encoded IGHV genes reflects the presence of more naïve B-cells in the parotid gland of pSS patients compared to the parotid gland of non-pSS sicca controls. With respect to the mutation frequency of the mutated sequences we observed that pSS IGHV sequences contained significantly (*p* < 0.0001) lower numbers of mutations than the non-pSS sicca sequences: 6.5% (IQR: 5.17–9.48% per IGHV sequence) and 7.3% (IQR: 4.17–8.62% per IGHV sequence), respectively.

### Increased Clonal Expansion of Ig-Producing Cells in Parotid Glands of pSS Patients

**Figure 3:**
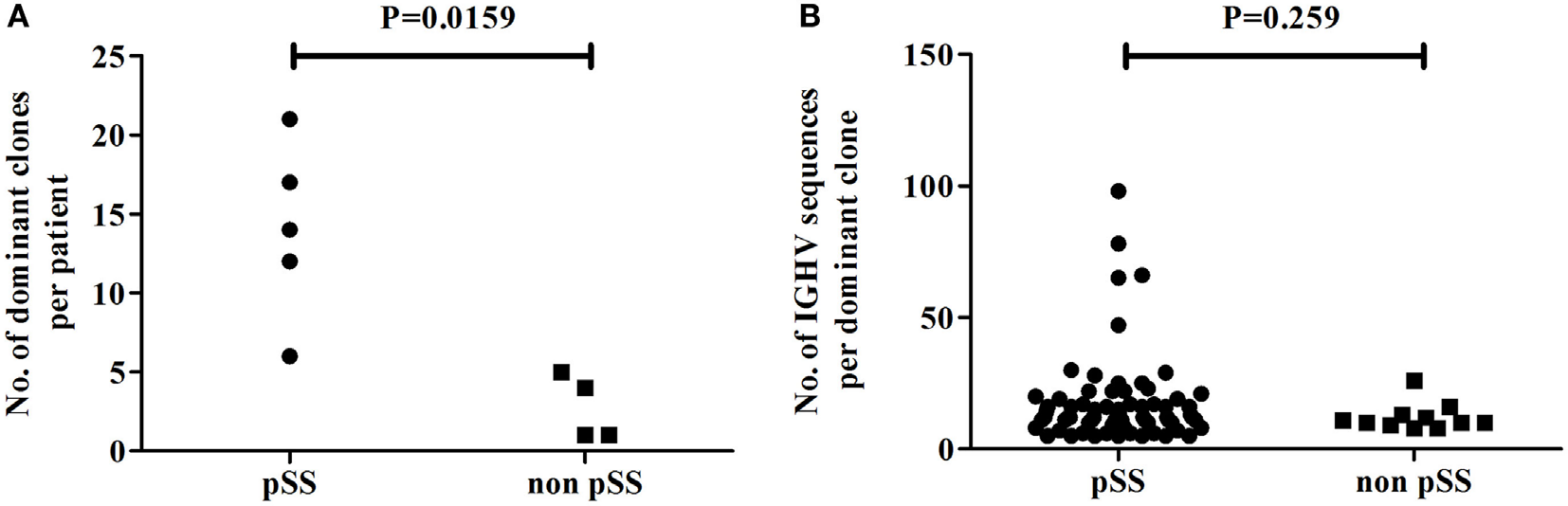
Clonal expansion of Ig-producing cells in the biopsies of the parotid salivary gland of pSS patients and non-pSS sicca controls. **(A)** Number of dominant clones present in the parotid gland per patient. **(B)** Number of IGHV sequences per dominant clone.

To compare the level of clonal expansion of Ig-producing cells in parotid glands of pSS patients and non-pSS sicca controls, we examined the number of dominant clones present in each sample. IGHV sequences were considered to be derived from clonally related cells when the nucleotide sequence of the CDR3 regions was identical and shared the same VH germline gene. The number of dominant clones in parotid gland biopsies was significantly higher in pSS patients, compared to non-pSS sicca controls (*p* = 0.0159) (Figure 3A). In pSS patients, there were between 6 and 22 dominant clones per biopsy, whereas in non-pSS sicca controls this was only between 2 and 4. In total, 70 dominant clones were observed in pSS parotid gland biopsies; whereas in non-pSS sicca biopsies, a total of 11 dominant clones was detected (Figure 3A). We did not see differences in clone size of these dominant clones between pSS patients and non-pSS sicca controls (Figure 3B).

### No Signs of Antigenic Selection Pressure on Ig-Producing Cells in the Parotid Gland of pSS Patients

**Figure 4:**
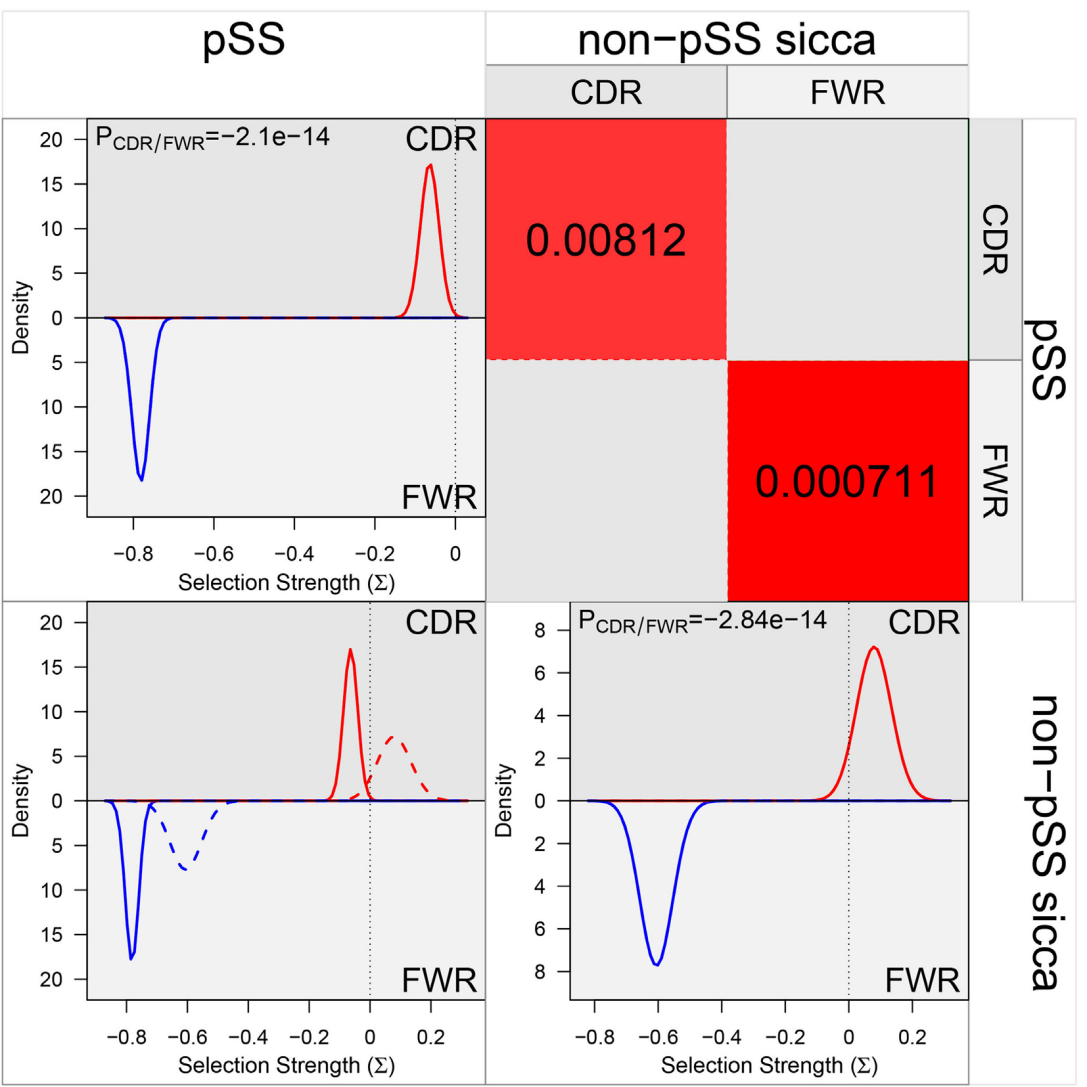
The selection pressures on IGHV sequences derived from dominant clones in pSS patients and non-pSS controls. All individual sequences from the dominant clones from pSS and non-pSS control parotid gland biopsies were inserted into the BASELINe program and the selection strength of both groups were compared. The figure is obtained as output data from the BASELINe program. The red line in the upper left plot represents the selection strength of the pSS sequences in the complementarity determining regions (CDRs). The blue line represents the (negative) selection strength in the framework (FWR) regions. In the lower right plot, the red line represents the selection strength of the non-pSS control sequences in the CDRs and the blue line the strength in the FWRs. The lower left plot is an overlay plot in which the solid line is the selection strength for pSS and the dashed line for non-pSS. Significance of differences in selection pressures was calculated both for CDRs and FWR regions between pSS and non-pSS samples in the upper right plot.

In order to study whether antigen selection plays a role in the development of the dominant clones, we used BASELINe. With this tool, possible antigen selection can be established based on the basis of the analysis of somatic mutation patterns (replacement versus silent mutations). Analysis of IGHV sequences derived from the dominant clones of the non-pSS sicca controls showed positive selection pressures in the CDRs (CDR1 and CDR2) and negative selection pressures in the FWRs (FWR1, FWR2, and FWR3) (Figure 4). Such a selection pressure profile is characteristic for antigen-driven clonal expansion. In marked contrast with the non-pSS sicca controls, significantly altered selection pressures were seen in IGHV sequences of dominant clones derived from pSS patients. The IGHV sequences of pSS patients demonstrated negative selection pressures in the CDRs, whereas selection pressures of the FWRs were even more negative than seen in non-pSS sicca controls (Figure 4). These data indicate that the formation of dominant Ig-producing clones in pSS patients is not typically antigen-driven and that alternative mechanisms might be involved in the establishment of clones in the salivary gland from pSS patients.

### Higher Incidence of Acquired N-Glycosylation Sites in IGHV Sequences of pSS Patients

**Figure 5:**
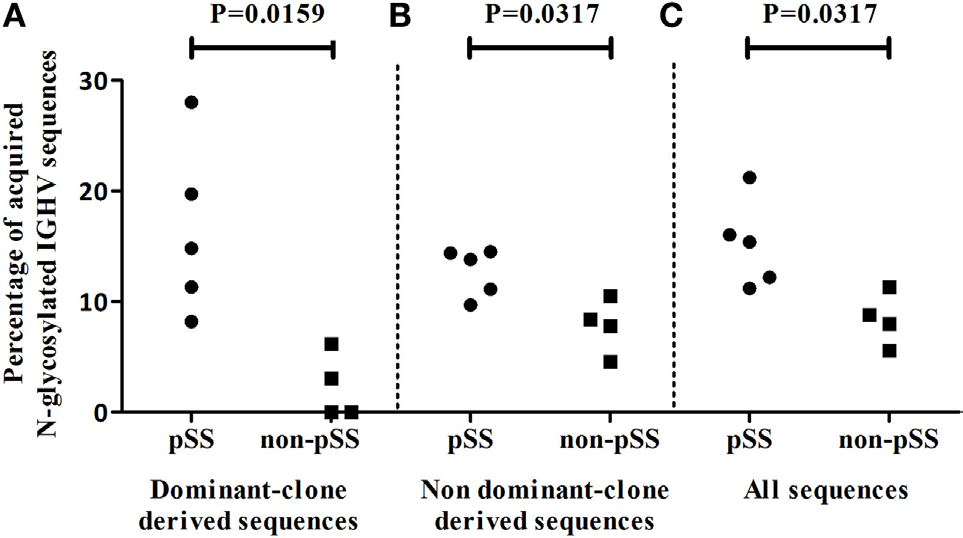
Incidence of ac-Nglycs in IGHV sequences in the salivary parotid gland biopsies. **(A)** The percentage of dominant clone-derived IGHV sequences containing at least one ac-Nglycs are shown per patient. **(B)** The percentage of glycosylated non-dominant clone-derived sequences per patient and **(C)** the percentage glycosylated sequences from all obtained IGHV (including dominant and non-dominant clone-derived IGHV sequences) sequences are depicted per patient.

A possible alternative selection mechanism that could be operational during B-cell expansion in the salivary gland of pSS patients could be lectin binding to glycosylated Ig (35). Glycosylation sites can be acquired during somatic hypermutation processes of IGHV genes (10, 25, 26). We, therefore, analyzed the presence of ac-Nglycs in the IGHV sequences from the dominant clones. The percentage of dominant-clone-derived sequences, containing at least one ac-Nglyc, was significantly higher (*p* = 0.0159) in pSS patients, compared to non-pSS sicca controls (Figure 5A; median pSS 14.8% IQR: 9.8–23.8%, median non-pSS sicca 1.6% IQR: 0.0–5.4%). In total, 180 IGHV sequences out of 1,223 (~15%) IGHV pSS sequences that belonged to dominant clones contained 189 ac-Nglycs (some sequences contained two or three ac-Nglycs); whereas in non-pSS sicca controls, this number was substantially lower: only 3 out of 133 (~2%) clonally related IGHV sequences contained ac-Nglycs.

To examine whether these Nglycs are restricted to IGHV sequences from dominant clones, we compared the percentage IGHV sequences containing an ac-Nglyc not belonging to the dominant clones obtained from pSS patient biopsies to those from non-pSS sicca control biopsies. Again, a significantly increased percentage (*p* = 0.0317) of N-glycosylated IGHV sequences in pSS was observed (Figure 5B; median pSS is 13.8% IQR: 10.4–14.4%, median non-pSS sicca is 8.1% IQR: 5.4–10.0%). When we did not discriminate between dominant and non-dominant clone-derived IGHV sequences, there still was a significant difference in percentage IGHV sequences with ac-Nglyc between pSS and non-pSS sicca (Figure 5C; median pSS is 15.4% IQR: 11.7–18.6%, median non-pSS sicca is 8.4% IQR: 6.2–10.7%). These findings suggest that Nglycs acquired during somatic hypermutation are not restricted to sequences from dominant clones.

### Preferential Acquired N-Glycosylation Sites in FWR3 Regions of IGHV Sequences from pSS Patients

Subsequently, we analyzed the location of the ac-Nglycs in the IGHV sequences derived from the dominant clones. Of the 180 IGHV sequences from pSS patient biopsies, 121 sequences were encoded by VH3 genes (67%), 56 sequences by VH4 genes (31%), and 3 sequences by VH5 genes (2%) in line with the different number of IGVH genes in the respective IGVH gene families. In non-pSS sicca controls, two of the IGHV sequences derived from dominant clones containing ac-Nglycs were encoded by VH3 genes and one by VH4. The majority of ac-Nglycs in pSS patients were the result of a replacement mutation in the FWR3 region: 115 out of 189 (61%) ac-Nglycs were located in the FWR3 region. The remaining 74 (39%) ac-Nglycs were located in the CDR compartment (CDR1 or CDR2). No ac-Nglycs were observed in FWR1 or FWR2. In the IGHV sequences derived from non-pSS sicca controls, all three ac-Nglycs were located in the CDRs (Table S1 in Supplementary Material).

Further analysis showed that most (66%) of the 84 ac-Nglycs in IGHV3 sequences were generated by an AA change at position 84 in the FWR3 region. There was also a high frequency (39%) of 22 ac-Nglycs in the FWR3 of IGHV4 sequences at AA position 90.

These data suggest the presence of hot-spots for ac-Nglycs in certain IGHV genes, particularly in the FWR3 region. At these sites, single change in nucleotide can lead to ac-Nglycs due to the composition of the flanking nucleotides.

### Naïve B-Cells Present in Dominant Clones of the Parotid Glands of pSS Patients

**Figure 6:**
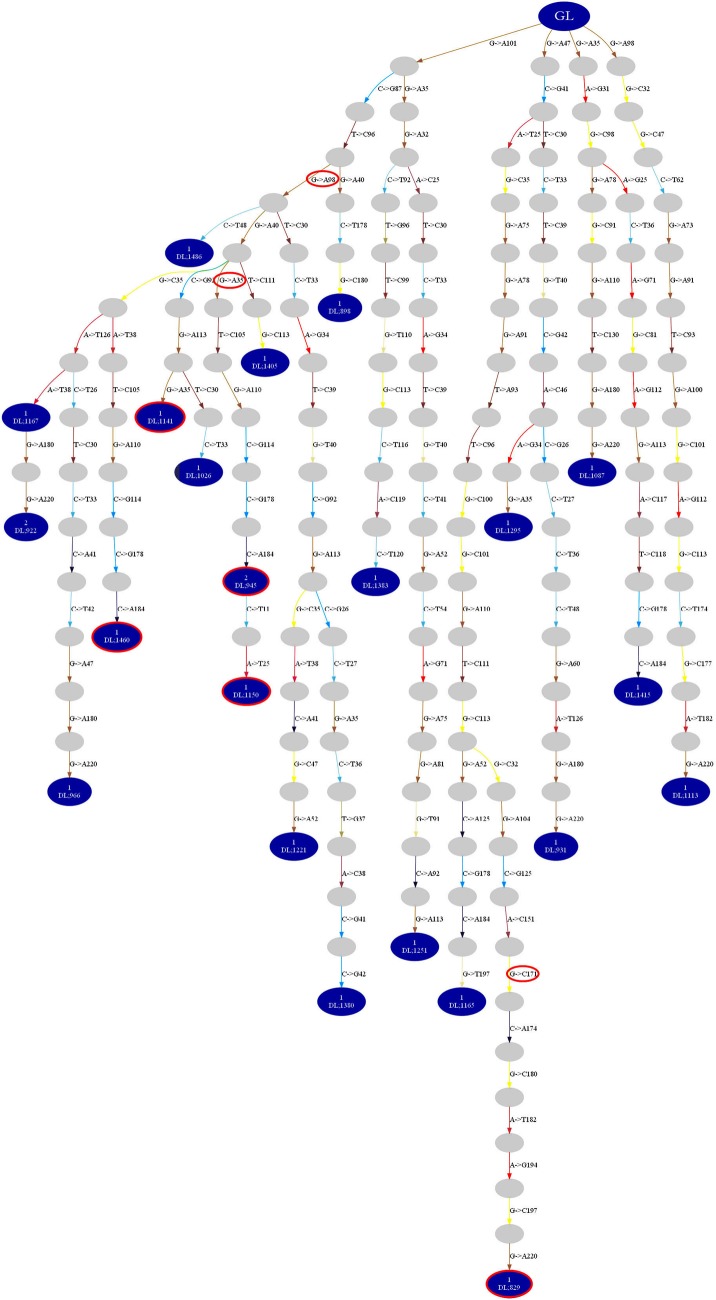
Lineage tree constructed by IgTree© from IGHV sequences of a dominant clone-derived from a parotid gland of a pSS patient. The blue dots depict the IGHV sequences obtained from the parotid gland biopsies. The gray dots represent theoretical IGHV sequences generated by the algorithm of the IgTree© program. The red circled dots represent obtained IGHV sequences with ac-Nglycs. The red circles depict the mutation responsible for ac-Nglycs. GL: germline sequence.

To study clonal evolution in relation to ac-Nglycs, the IGHV sequences belonging to a dominant clone were implemented into the algorithm of IgTree©. Mutations leading to new ac-Nglycs could be found at every level of the lineage tree constructed from the clonally related IGHV sequences of the various dominant clones from the pSS patients; an example of a lineage tree of a dominant clone-derived from a parotid gland of a pSS patient is shown in Figure 6. This observation suggests that mutations leading to ac-Nglycs are not a necessary event for clonal expansion.

Analysis of the lineage trees also showed that in four out five pSS patients, a clone was found that comprised the original, unmutated, germline sequence among the observed IGHV sequences. Such clones were not observed in non-pSS sicca controls. The presence of both the germline root sequence and mutated IGHV sequences within the same dominant clone provide evidence that this clone developed from a naïve B-cell that was recruited into the parotid gland where it underwent local expansion and somatic hypermutation. We found no differences in trunk length and path length indicating that there is no difference in affinity and selection threshold in the lineage trees generated from dominant clones of pSS compared to non-pSS sicca (data not shown). Based on these two parameters, we could not discriminate in the diversification history between pSS and non-pSS.

### Stereotypic RF Rearrangements Present in IGHV Sequences from Parotid Gland of pSS Patients

Parotid gland MALT lymphomas frequently express BCRs, characterized by a striking AA sequence homology to the IGHV-CDR3 of stereotypic RFs. These RFs are encoded by typical combinations of restricted IGHV and IG kappa V gene rearrangements. Here, we focused on the IGHV rearrangements. Five groups of somatically mutated stereotypic RF BCRs have been identified (11, 31, 32). Stereotypic RF rearrangements were not found in dominant clones from both pSS and non-pSS sicca. Analysis of all IGHV sequences derived from the parotid glands of pSS and non-pSS sicca revealed only very few stereotypic RF sequences in three out of five pSS patients and none in non-pSS sicca controls. Both in pSS patient 3 (pSS3) and 4 (pSS4), VH3-7/JH3 rearrangements were found with CDR3s that were homologous to stereotypic to V3-7-RFs. In pSS3, 31 of 4,039 IGHV sequences were encoded by this rearrangement. Within these 31 sequences, a total of 4 sets of clonally related sequences were detected with 2, 4, 8, and 10 members, respectively. The CDR3 regions of all these sequences were nearly identical, with differences ranging from one to three AAs, suggesting that the 31 sequences probably belong to the same clone. In the biopsy of patient pSS4, 5 of 3,229 IGHV sequences harbored the VH3-7/JH3 rearrangement, homologous to V3-7-RF. These five sequences exhibited some minor differences in the CDR3 regions, and thus also may belong to a single clone. Patient pSS5 harbored only one IGHV sequence with a VH1-69/JH4 rearrangement that was homologous to stereotypic WOL-RFs. None of the IGHV sequences that were homologous to stereotypic RFs contained ac-Nglycs. The data indicate that stereotypic RFs are occasionally present in parotid glands of pSS patients.

## Discussion

With NGS technology, we showed, in agreement with others, that large numbers of Ig-producing clones are present in the glandular tissue (6, 7, 9, 36, 37) of pSS patients reflecting the characteristic B-cell hyperactivity in these patients (1). Importantly, with NGS, we were able to demonstrate now the presence of unmutated root/founder sequence from which dominant clones arise in the parotid gland of pSS patients. Another main finding was that we found no indications that antigen selection plays a role in generation of these clones. We did, however, observe an increase in the number of ac-Nglycs in both clonally related and not clonally related IGHV transcripts of pSS patients, as a result of the somatic hypermutations. Thus, alternative driving forces and/or selection mechanisms might be involved in the survival and expansion of B-cells in the development of pSS.

### Origin of B-Cell Clones in the Parotid Gland

Little is known about the origin of the large number of B-cell clones found in parotid glands of pSS patients. One possible explanation is that these clones arise from CD27+ memory B-cells that enter the salivary glands, where they subsequently clonally expand (37, 38). We observed that in the parotid glands of pSS patients (but not in non-pSS sicca controls) dominant clones were present that were comprised of both unmutated (germline) IGHV sequences and somatically hypermutated sequences. The unmutated sequence is most likely derived from a naïve B-cell that migrated into the parotid gland. Here, the naïve B-cell underwent local proliferation and somatic hypermutation, leading to the formation of a clone. Naïve IGHV sequences were significantly less seen in the parotid gland biopsies of non-pSS sicca controls (Figure 2). The presence of only mutated IGHV sequences in the dominant clones from non-pSS sicca controls suggests local expansion from memory cells. However, due to the small amount of dominant clones in non-pSS sicca controls, we cannot completely exclude the possibility of clonal expansion from naïve B-cells in non-pSS sicca controls. Just as we cannot exclude that in pSS patients part of the clones still might arise from mutated memory cells that enter the parotid gland and expand.

An obvious site for this expansion and somatic hypermutation are the (ectopic) GCs that can be present in the periductal infiltrates of the salivary glands. Similar to regular GCs in peripheral lymphoid organs, these ectopic GCs are composed of activated B-cells, T-cells, and follicular dendritic cell networks (1, 4, 39–41). Whether GCs are involved in the formation of dominant clones in parotid glands is, however, questionable. First, only a minor proportion (~25%) of the biopsies of pSS patients exhibit GCs in the periductal infiltrate (4), whereas all pSS patients exhibit clonally expanded Ig-producing clones. Histopathological examination of pSS parotid biopsies revealed GCs in only one of the five patients studied. Although not formally proven, it is thus rather unlikely that GCs are involved in formation of dominant clones in parotid glands of pSS patients. Second, somatic hypermutation in classical GCs is associated with stringent antigen selection guided by immune complexes trapped on the surface of follicular dendritic cells (42). Analysis of the mutation patterns (in terms of silent versus replacement mutations) of the IGHV sequences derived from dominant clones in pSS parotid glands did not show any evidence for antigen-driven selection of Ig-sequences. Apparently, at least part of the Ig-producing clones is derived from naïve B-cells that are recruited to the salivary gland during inflammation, where they are activated, proliferate, somatically hypermutate, and undergo isotype switching in a GC-independent fashion in the absence of antigen-driven selection. This is in marked contrast to non-pSS sicca controls. The IGHV sequences of dominant clones in these disease controls are clearly the result of a classical antigen-selection process. However, GCs are absent in the non-pSS sicca control salivary glands and no clonally related unmutated sequences are found. A plausible explanation for this might be that isotype (IgA) switched CD27+ memory B-cells are formed in a classical way in GCs located at distal, mucosal sites. These pre-selected memory cells might enter the parotid gland as IgA+ memory B-cells to further proliferate during which they acquire some additional mutations, and finally differentiate to plasmablasts and plasmacells. This scenario resembles what has been proposed for gut IgA plasma cells in humans (43).

### Increased Acquired N-Glycosylation Sites in IGHV Sequences in pSS

We did not find indications for signs of antigen selection in the dominant clones in pSS patients. However, we observed a significantly higher incidence of ac-Nglycs in the IGHV sequences derived from the dominant clones in parotid gland tissue from pSS patients compared to non-pSS sicca controls. We observed that the majority of the ac-Nglycs were present in certain hot-spots, in particular in the FWR3 region of IGHV3 and IGHV4 sequences. A single nucleotide change at this spot during the somatic hypermutation process could already result in ac-Nglycs. The increased frequency of ac-Nglycs is, however, not restricted for IGHV sequences of the dominant clones. The higher incidence of ac-Nglycs seems to be a common phenomenon in the total pool of IGHV sequences obtained from the salivary gland biopsies of pSS patients (Figure 5). Moreover, ac-Nglycs have been found in RA (26) and other non-rheumatic autoimmune diseases. We even obtained evidence in a meta-analysis of published IGVH genes from patients with autoimmune diseases and controls that acquiring of new Nglycs in variable regions of immunoglobulin genes is a common feature in patients with autoimmune diseases (25). The relevance of this Fab glycosylation in autoimmunity still needs to be further investigated.

Fc-glycosylated antibodies have been found in anti-neutrophil cytoplasmic antibody (ANCA)-associated systemic vasculitis (AAV), SLE, pSS, and RA. Fc-glycosylation patterns were associated with disease activity and severity and outcome (44–49). In RA also glycosylated Fab antibodies have been found (44, 50–52). Glycosylation of the Fab fragment of Ig can influence the affinity for antigens (53, 54), but it also has been postulated in AAV that Fab glycosylation can have effector functions. Highly Fab glycosylated ANCA IgG had an increased ability to induce the respiratory burst in neutrophils *in vitro* compared to the non-glycosylated ANCA IgG. However, the role of Fc glycosylation cannot completely be excluded in this assay (50). Sometimes Fab glycosylation even can correlate with disease activity (44, 50). Of note, the described effects of glycosylation are at the protein level and the ac-Nglycs in our NGS study are at transcription level and, therefore, potential sites for glycosylation. A major difference between Fc and Fab glycosylation is that Fc glycosylation is genetically encoded while Fab glycosylation seems to be introduced as a result of somatic hypermutation of the IGV-region.

The introduction of new Nglycs in the IGHV sequences in pSS may offer Ig expressing cells an alternative selection mechanism to escape from the classical selection mechanism in GCs. This is strengthened by lack of evidence for antigen-driven selection of the IGHV sequences and the explicit tendency for ac-Nglycs to occur within the FWRs. Binding of the glycosylated BCR with glycan receptors, such as lectins, in the vicinity of the receptor could rescue B-cells from cell death. Coelho et al. (35) demonstrated that recombinant lectin domains of the mannose receptor or DC-SIGN binds *in vitro* to mannosylated Igs of follicular lymphoma cells to trigger BCR-mediated signaling events (Ca2+flux). Binding of lectins to glycosylated Igs was presumed to lead to survival and even to expansion of lymphoma cells. Lectins are widespread and are both expressed by many cell types, such as T-cells, NK cells, macrophages, dendritic cells, and by micro-organisms (55–58). This makes inflamed parotid tissue the ideal microenvironment for lectins to bind to the glycosylated Igs.

### Rheumatoid Factor

The increased capacity of clonal expansion of B-cells in the exocrine glands of pSS patients significantly increases the risk of developing MALT-lymphoma (5, 59, 60). A characteristic feature of MALT-lymphoma is the expression of BCR with RF reactivity. It has been shown by Bende et al. (31) that at least 40% of the cases of salivary gland MALT lymphoma, express BCRs with strong CDR3 homology to RFs (stereotypic RFs). They further showed that these stereotypic RF clones were only sporadically present in SS labial salivary glands (11). Analysis of our parotid gland sequence data confirmed the observations in the labial salivary glands (11). Although we observed some stereotypic RF rearrangements in three out of five pSS patients RF rearrangements were not observed in any of the dominant clones. The observation of a relative small number of sequences with stereotypic RF in the parotid gland tissue of pSS patients suggest that antigen-specific triggers are needed for these rare B-cells to expand and transform to neoplastic MALT lymphoma cells.

In summary, we demonstrate that at least part of the Ig-producing clones within the pSS glands are the result of local proliferation of naïve unmutated B-cells. The high number of Nglycs, acquired in the IGHV sequences during somatic hypermutation, may generate a selective advantage for Ig-producing B-cells in the parotid gland tissue to survive selection mechanisms. The interactions of the (potential) glycans with lectins in the environment of the inflamed parotid gland tissue could offer (autoreactive) B-cells this alternative selection mechanism.

## Ethics Statement

Parotid biopsies were originally collected for diagnostic purposes. Usage of these biopsies for research purposes was obtained on written informed consent with approval from the medical ethical committee of the University Medical Center Groningen, Groningen, the Netherlands.

## Author Contributions

FK, HB, and NB designed the study. HB and ArV recruited the patients and provided clinical data. FS performed the surgical procedures. MD and NV performed/supervised the NGS and the initial screening of the data. AnV performed further analysis of the NGS data and wrote the concept of this manuscript. RB performed the stereotypic RF analysis. NB and FK were involved in writing and discussing the data. All authors critically reviewed the manuscript and approved the final version to be published.

## Conflict of Interest Statement

The authors declare that the research was conducted in the absence of any commercial or financial relationships that could be construed as a potential conflict of interest.

## Footnotes

^1^http://www.imgt.org.

^2^https://blast.ncbi.nlm.nih.gov/Blast.cgi.

^3^http://clustalw.ddbj.nig.ac.jp.

^4^http://www.cbs.dtu.dk/services/NetNGlyc.

## Supplementary Material

Table S1 Ac-Nglycs observed in the IGHV sequences from dominant clones per patient. In this table, the incidence of ac-Nglycs and the position (AA) of these sites in the IGHV sequences of each dominant clones are depicted. Between brackets, the number of times ac-Nglycs are present at that position is given.

## References

[1] Kroese FG Abdulahad WH Haacke E Bos NA Vissink A Bootsma H B-cell hyperactivity in primary Sjögren’s syndrome. Expert Rev Clin Immunol (2014) 10:483–99. 10.1586/1744666X.2014.891439 24564507

[2] Hernández-Molina G Leal-Alegre G Michel-Peregrina M The meaning of anti-Ro and anti-La antibodies in primary Sjögren’s syndrome. Autoimmun Rev (2011) 10:123–5. 10.1016/j.autrev.2010.09.001 20833272

[3] Ramos-Casals M Solans R Rosas J Camps MT Gil A Del Pino-Montes J Primary Sjögren syndrome in Spain: clinical and immunologic expression in 1010 patients. Medicine (Baltimore) (2008) 87:210–9. 10.1097/MD.0b013e318181e6af 18626304

[4] Jonsson MV Skarstein K Jonsson R Brun JG Serological implications of germinal center-like structures in primary Sjögren’s syndrome. J Rheumatol (2007) 34:2044–9. 17787040

[5] Giannouli S Voulgarelis M Predicting progression to lymphoma in Sjögren’s syndrome patients. Expert Rev Clin Immunol (2014) 10:501–12. 10.1586/1744666X.2014.872986 24451006

[6] Stott DI Hiepe F Hummel M Steinhauser G Berek C Antigen-driven clonal proliferation of B cells within the target tissue of an autoimmune disease. The salivary glands of patients with Sjögren’s syndrome. J Clin Invest (1998) 102:938–46. 10.1172/JCI3234 9727062 PMC508959

[7] Gellrich S Rutz S Borkowski A Golembowski S Gromnica-Ihle E Sterry W Analysis of VH-D-JH gene transcripts in B cells infiltrating the salivary glands and lymph node tissues of patients with Sjögren’s syndrome. Arthritis Rheum (1999) 42:240–7. 10.1002/1529-0131(199902)42:2<240::AID-ANR5>3.0.CO;2-I 10025917

[8] Hansen A Jacobi A Pruss A Kaufmann O Scholze J Lipsky PE Comparison of immunoglobulin heavy chain rearrangements between peripheral and glandular B cells in a patient with primary Sjögren’s syndrome. Scand J Immunol (2003) 57:470–9. 10.1046/j.1365-3083.2003.01226.x 12753504

[9] Hamza N Bootsma H Yuvaraj S Spijkervet FKL Haacke EA Pollard RPE Persistence of immunoglobulin-producing cells in parotid salivary glands of patients with primary Sjögren’s syndrome after B cell depletion therapy. Ann Rheum Dis (2012) 71:1881–7. 10.1136/annrheumdis-2011-201189 22615459

[10] Hamza N Hershberg U Kallenberg CGM Vissink A Spijkervet FKL Bootsma H Ig gene analysis reveals altered selective pressures on Ig-producing cells in parotid glands of primary Sjögren’s syndrome patients. J Immunol (2015) 194:514–21. 10.4049/jimmunol.1302644 25488989

[11] Bende RJ Slot LM Hoogeboom R Wormhoudt TAM Adeoye AO Guikema JEJ Stereotypic rheumatoid factors that are frequently expressed in mucosa-associated lymphoid tissue-type lymphomas are rare in the labial salivary glands of patients with Sjögren’s syndrome. Arthritis Rheumatol (2015) 67:1074–83. 10.1002/art.39002 25546553

[12] Corneth OBJ Verstappen GMP Paulissen SMJ de Bruijn MJW Rip J Lukkes M Enhanced Bruton’s tyrosine kinase activity in peripheral blood B lymphocytes of autoimmune disease patients. Arthritis Rheumatol (2017) 69(6):1313–24. 10.1002/art.40059 28141917

[13] Groom J Kalled SL Cutler AH Olson C Woodcock SA Schneider P Association of BAFF/BLyS overexpression and altered B cell differentiation with Sjögren’s syndrome. J Clin Invest (2002) 109:59–68. 10.1172/JCI0214121 11781351 PMC150825

[14] Pers J-O Daridon C Devauchelle V Jousse S Saraux A Jamin C BAFF overexpression is associated with autoantibody production in autoimmune diseases. Ann N Y Acad Sci (2005) 1050:34–9. 10.1196/annals.1313.004 16014518

[15] Pers J-O d’Arbonneau F Devauchelle-Pensec V Saraux A Pennec Y-L Youinou P Is periodontal disease mediated by salivary BAFF in Sjögren’s syndrome? Arthritis Rheum (2005) 52:2411–4. 10.1002/art.21205 16052575

[16] Jonsson MV Szodoray P Jellestad S Jonsson R Skarstein K Association between circulating levels of the novel TNF family members APRIL and BAFF and lymphoid organization in primary Sjögren’s syndrome. J Clin Immunol (2005) 25:189–201. 10.1007/s10875-005-4091-5 15981083

[17] Pollard RPE Abdulahad WH Vissink A Hamza N Burgerhof JGM Meijer JM Serum levels of BAFF, but not APRIL, are increased after rituximab treatment in patients with primary Sjogren’s syndrome: data from a placebo-controlled clinical trial. Ann Rheum Dis (2013) 72:146–8. 10.1136/annrheumdis-2012-202071 22851468

[18] Mariette X Roux S Zhang J Bengoufa D Lavie F Zhou T The level of BLyS (BAFF) correlates with the titre of autoantibodies in human Sjögren’s syndrome. Ann Rheum Dis (2003) 62:168–71. 10.1136/ard.62.2.168 12525388 PMC1754442

[19] Kang KY Kim H-O Kwok S-K Ju JH Park K-S Sun D-I Impact of interleukin-21 in the pathogenesis of primary Sjögren’s syndrome: increased serum levels of interleukin-21 and its expression in the labial salivary glands. Arthritis Res Ther (2011) 13:R179 10.1186/ar3504 22030011 PMC3308114

[20] Szodoray P Alex P Brun JG Centola M Jonsson R Circulating cytokines in primary Sjögren’s syndrome determined by a multiplex cytokine array system. Scand J Immunol (2004) 59:592–9. 10.1111/j.0300-9475.2004.01432.x 15182255

[21] Pollard RPE Abdulahad WH Bootsma H Meiners PM Spijkervet FKL Huitema MG Predominantly proinflammatory cytokines decrease after B cell depletion therapy in patients with primary Sjogren’s syndrome. Ann Rheum Dis (2013) 72:2048–50. 10.1136/annrheumdis-2013-203447 23864239

[22] Zhu D McCarthy H Ottensmeier CH Johnson P Hamblin TJ Stevenson FK. Acquisition of potential N-glycosylation sites in the immunoglobulin variable region by somatic mutation is a distinctive feature of follicular lymphoma. Blood (2002) 99:2562–8. 10.1182/blood.V99.7.2562 11895794

[23] Zabalegui N de Cerio AL-D Inogés S Rodríguez-Calvillo M érez-Calvo JP Hernández M Acquired potential N-glycosylation sites within the tumor-specific immunoglobulin heavy chains of B-cell malignancies. Haematologica (2004) 89:541–6. 15136216

[24] Fenwick MK Escobedo FA. Exploration of factors affecting the onset and maturation course of follicular lymphoma through simulations of the germinal center. Bull Math Biol (2009) 71:1432–62. 10.1007/s11538-009-9408-8 19412639

[25] Visser A Hamza N Kroese FGM Bos NA Acquiring new N-glycosylation sites in variable regions of immunoglobulin genes by somatic hypermutation is a common feature of autoimmune diseases. Ann Rheum Dis (2017). 10.1136/annrheumdis-2017-212568 29102958

[26] Vergroesen RD Slot LM Hafkenscheid L Koning MT van der Voort EIH Grooff CA B-cell receptor sequencing of anti-citrullinated protein antibody (ACPA) IgG-expressing B cells indicates a selective advantage for the introduction of *N*-glycosylation sites during somatic hypermutation. Ann Rheum Dis (2017). 10.1136/annrheumdis-2017-212052 28835463

[27] Shiboski CH Shiboski SC Seror R Criswell LA Labetoulle M Lietman TM American college of rheumatology/European league against rheumatism classification criteria for primary Sjögren’s syndrome: a consensus and data-driven methodology involving three international patient cohorts. Arthritis Rheumatol (2016) 69:35–45. 10.1002/art.39859 27785888 PMC5650478

[28] Spijkervet FKL Haacke E Kroese FGM Bootsma H Vissink A. Parotid gland biopsy, the alternative way to diagnose Sjögren syndrome. Rheum Dis Clin North Am (2016) 42:485–99. 10.1016/j.rdc.2016.03.007 27431350

[29] Maillette de Buy Wenniger LJ Doorenspleet ME Klarenbeek PL Verheij J Baas F Elferink RPO Immunoglobulin G4+ clones identified by next-generation sequencing dominate the B cell receptor repertoire in immunoglobulin G4 associated cholangitis. Hepatology (2013) 57:2390–8. 10.1002/hep.26232 23300096

[30] Doorenspleet ME Klarenbeek PL de Hair MJH van Schaik BDC Esveldt REE van Kampen AHC Rheumatoid arthritis synovial tissue harbours dominant B-cell and plasma-cell clones associated with autoreactivity. Ann Rheum Dis (2014) 73:756–62. 10.1136/annrheumdis-2012-202861 23606709

[31] Bende RJ Aarts WM Riedl RG de Jong D Pals ST van Noesel CJM Among B cell non-Hodgkin’s lymphomas, MALT lymphomas express a unique antibody repertoire with frequent rheumatoid factor reactivity. J Exp Med (2005) 201:1229–41. 10.1084/jem.20050068 15837810 PMC2213160

[32] Bende RJ Janssen J Wormhoudt TAM Wagner K Guikema JEJ van Noesel CJM Identification of a novel stereotypic IGHV4-59/IGHJ5-encoded B-cell receptor subset expressed by various B-cell lymphomas with high affinity rheumatoid factor activity. Haematologica (2016) 101:e200–3. 10.3324/haematol.2015.139626 26858354 PMC5004362

[33] Barak M Zuckerman NS Edelman H Unger R Mehr R IgTree©: creating immunoglobulin variable region gene lineage trees. J Immunol Methods (2008) 338:67–74. 10.1016/j.jim.2008.06.006 18706908

[34] Yaari G Uduman M Kleinstein SH. Quantifying selection in high-throughput Immunoglobulin sequencing data sets. Nucleic Acids Res (2012) 40:e134. 10.1093/nar/gks457 22641856 PMC3458526

[35] Coelho V Krysov S Ghaemmaghami AM Emara M Potter KN Johnson P Glycosylation of surface Ig creates a functional bridge between human follicular lymphoma and microenvironmental lectins. Proc Natl Acad Sci U S A (2010) 107:18587–92. 10.1073/pnas.1009388107 20937880 PMC2972945

[36] Hansen A Gosemann M Pruss A Reiter K Ruzickova S Lipsky PE Abnormalities in peripheral B cell memory of patients with primary Sjögren’s syndrome. Arthritis Rheum (2004) 50:1897–908. 10.1002/art.20276 15188366

[37] Hansen A Odendahl M Reiter K Jacobi AM Feist E Scholze J Diminished peripheral blood memory B cells and accumulation of memory B cells in the salivary glands of patients with Sjögren’s syndrome. Arthritis Rheum (2002) 46:2160–71. 10.1002/art.10445 12209521

[38] Aqrawi LA Brokstad KA Jakobsen K Jonsson R Skarstein K Low number of memory B cells in the salivary glands of patients with primary Sjögren’s syndrome. Autoimmunity (2012) 45:547–55. 10.3109/08916934.2012.712170 22849322

[39] Salomonsson S Jonsson MV Skarstein K Brokstad KA Hjelmström P Wahren-Herlenius M Cellular basis of ectopic germinal center formation and autoantibody production in the target organ of patients with Sjögren’s syndrome. Arthritis Rheum (2003) 48:3187–201. 10.1002/art.11311 14613282

[40] Risselada AP Looije MF Kruize AA Bijlsma JWJ van Roon JAG The role of ectopic germinal centers in the immunopathology of primary Sjögren’s syndrome: a systematic review. Semin Arthritis Rheum (2013) 42:368–76. 10.1016/j.semarthrit.2012.07.003 22995442

[41] Le Pottier L Devauchelle V Fautrel A Daridon C Saraux A Youinou P Ectopic germinal centers are rare in Sjogren’s syndrome salivary glands and do not exclude autoreactive B cells. J Immunol (2009) 182:3540–7. 10.4049/jimmunol.0803588 19265132

[42] MacLennan ICM. Germinal centers. Annu Rev Immunol (1994) 12:117–39. 10.1146/annurev.iy.12.040194.001001 8011279

[43] Yuvaraj S Dijkstra G Burgerhof JGM Dammers PM Stoel M Visser A Evidence for local expansion of IgA plasma cell precursors in human ileum. J Immunol (2009) 183:4871–8. 10.4049/jimmunol.0901315 19786537

[44] Holland M Yagi H Takahashi N Kato K Savage COS Goodall DM Differential glycosylation of polyclonal IgG, IgG-Fc and IgG-Fab isolated from the sera of patients with ANCA-associated systemic vasculitis. Biochim Biophys Acta (2006) 1760:669–77. 10.1016/j.bbagen.2005.11.021 16413679

[45] Holland M Takada K Okumoto T Takahashi N Kato K Adu D Hypogalactosylation of serum IgG in patients with ANCA-associated systemic vasculitis. Clin Exp Immunol (2002) 129:183–90. 10.1046/j.1365-2249.2002.01864.x 12100039 PMC1906423

[46] Magorivska I Muñoz LE Janko C Dumych T Rech J Schett G Sialylation of anti-histone immunoglobulin G autoantibodies determines their capabilities to participate in the clearance of late apoptotic cells. Clin Exp Immunol (2016) 184:110–7. 10.1111/cei.12744 26618514 PMC4778101

[47] Sjöwall C Zapf J von Löhneysen S Magorivska I Biermann M Janko C Altered glycosylation of complexed native IgG molecules is associated with disease activity of systemic lupus erythematosus. Lupus (2015) 24:569–81. 10.1177/0961203314558861 25389233

[48] Ercan A Cui J Chatterton DEW Deane KD Hazen MM Brintnell W Aberrant IgG galactosylation precedes disease onset, correlates with disease activity, and is prevalent in autoantibodies in rheumatoid arthritis. Arthritis Rheum (2010) 62:2239–48. 10.1002/art.27533 20506563 PMC4118465

[49] Rombouts Y Ewing E van de Stadt LA Selman MHJ Trouw LA Deelder AM Anti-citrullinated protein antibodies acquire a pro-inflammatory Fc glycosylation phenotype prior to the onset of rheumatoid arthritis. Ann Rheum Dis (2015) 74:234–41. 10.1136/annrheumdis-2013-203565 24106048

[50] Xu P-C Gou S-J Yang X-W Cui Z Jia X-Y Chen M Influence of variable domain glycosylation on anti-neutrophil cytoplasmic autoantibodies and anti-glomerular basement membrane autoantibodies. BMC Immunol (2012) 13:10. 10.1186/1471-2172-13-10 22404873 PMC3324382

[51] Youings A Chang SC Dwek RA Scragg IG. Site-specific glycosylation of human immunoglobulin G is altered in four rheumatoid arthritis patients. Biochem J (1996) 314(Pt 2):621–30. 10.1042/bj3140621 8670078 PMC1217093

[52] Rombouts Y Willemze A van Beers JJBC Shi J Kerkman PF van Toorn L Extensive glycosylation of ACPA-IgG variable domains modulates binding to citrullinated antigens in rheumatoid arthritis. Ann Rheum Dis (2016) 75:578–85. 10.1136/annrheumdis-2014-206598 25587188

[53] Coloma MJ Trinh RK Martinez AR Morrison SL Position effects of variable region carbohydrate on the affinity and in vivo behavior of an anti-(1 –> 6) dextran antibody. J Immunol (1999) 162:2162–70. 9973491

[54] Wright A Tao MH Kabat EA Morrison SL. Antibody variable region glycosylation: position effects on antigen binding and carbohydrate structure. EMBO J (1991) 10:2717–23. 1717254 10.1002/j.1460-2075.1991.tb07819.x PMC452979

[55] Park SM Angel CE McIntosh JD Mansell CM Chen C-JJ Cebon J Mapping the distinctive populations of lymphatic endothelial cells in different zones of human lymph nodes. PLoS One (2014) 9:e94781. 10.1371/journal.pone.0094781 24733110 PMC3986404

[56] Linley A Krysov S Ponzoni M Johnson PW Packham G Stevenson FK. Lectin binding to surface Ig variable regions provides a universal persistent activating signal for follicular lymphoma cells. Blood (2015) 126:1902–10. 10.1182/blood-2015-04-640805 26194765

[57] Tellier J Menard C Roulland S Martin N Monvoisin C Chasson L Human t(14;18)positive germinal center B cells: a new step in follicular lymphoma pathogenesis? Blood (2014) 123:3462–5. 10.1182/blood-2013-12-545954 24677543

[58] Sungalee S Mamessier E Morgado E Grégoire E Brohawn PZ Morehouse CA Germinal center reentries of BCL2-overexpressing B cells drive follicular lymphoma progression. J Clin Invest (2014) 124:5337–51. 10.1172/JCI72415 25384217 PMC4348942

[59] Voulgarelis M Ziakas PD Papageorgiou A Baimpa E Tzioufas AG Moutsopoulos HM Prognosis and outcome of non-Hodgkin lymphoma in primary Sjögren syndrome. Medicine (Baltimore) (2012) 91:1–9. 10.1097/MD.0b013e31824125e4 22198497

[60] Nocturne G Boudaoud S Miceli-Richard C Viengchareun S Lazure T Nititham J Germline and somatic genetic variations of TNFAIP3 in lymphoma complicating primary Sjogren’s syndrome. Blood (2013) 122:4068–76. 10.1182/blood-2013-05-503383 24159176 PMC3862283

